# Time-Based Stress and Procedural Justice: Can Transparency Mitigate the Effects of Algorithmic Compensation in Gig Work?

**DOI:** 10.3390/ijerph21010086

**Published:** 2024-01-11

**Authors:** Benjamin Semujanga, Xavier Parent-Rocheleau

**Affiliations:** Department of Human Resources Management, HEC Montréal, 3000 Côte Ste-Catherine, Montréal, QC H3T 2A7, Canada; xavier.parent-rocheleau@hec.ca

**Keywords:** algorithmic management, algorithmic compensation, procedural justice, time-based stress, algorithmic transparency, gig work, gig economy, online labor platforms

## Abstract

The gig economy has led to a new management style, using algorithms to automate managerial decisions. Algorithmic management has aroused the interest of researchers, particularly regarding the prevalence of precarious working conditions and the health issues related to gig work. Despite algorithmically driven remuneration mechanisms’ influence on work conditions, few studies have focused on the compensation dimension of algorithmic management. We investigate the effects of algorithmic compensation on gig workers in relation to perceptions of procedural justice and time-based stress, two important predictors of work-related health problems. Also, this study examines the moderating effect of algorithmic transparency in these relationships. Survey data were collected from 962 gig workers via a research panel. The results of hierarchical multiple regression analysis show that the degree of exposure to algorithmic compensation is positively related to time-based stress. However, contrary to our expectations, algorithmic compensation is also positively associated with procedural justice perceptions and our results indicate that this relation is enhanced at higher levels of perceived algorithmic transparency. Furthermore, transparency does not play a role in the relationship between algorithmic compensation and time-based stress. These findings suggest that perceived algorithmic transparency makes algorithmic compensation even fairer but does not appear to make it less stressful.

## 1. Introduction

In recent years, the labor market has been going through a period of radical change, characterized by the introduction of new technologies in businesses, ushering in a fourth industrial revolution [1,2]. One of the distinctive features of this revolution consists of the increasing number of organizations that use algorithms in their decision-making processes, automating tasks and responsibilities typically performed by managers. This refers to algorithmic management (AM), a process aimed at automating managerial decisions or assisting managers with algorithms [3,4,5]. While its use is increasing in traditional industries, this new form of management is prevalent in the gig economy, of which Uber is the most well-known platform, and where most of the operational management is performed using algorithms [5,6,7,8]. Platforms using this disruptive technology are found in numerous sectors such as Upwork for freelance tasks; Task Rabbit for home services; or Amazon Mechanical Turk (MTurk), Microworkers and Clickworker for microtasks [5,9,10]. Moreover, due to the rise of the gig economy, this novel management method is experiencing notable growth, becoming a global phenomenon attracting millions of workers and consumers. In fact, while estimates vary, there are roughly 70 million workers and this number is estimated to grow at a 26% annual pace [11,12]. Furthermore, it is estimated 8% of workers in the Canada participate in the gig economy, a smaller number is found in the EU, UK and US with 3%, 4% and 4.5%, respectively [9,13]. Also, India is estimated to have more than 3 million gig economy workers [14].

This rise had led researchers in different disciplines to study the impacts of this new business model, especially with a focus on people who use these platforms to earn an income, called gig workers. Currently, the literature is struggling to catch up with real-world applications of AM [6]. Nevertheless, the literature highlights more unfavorable than positive effects for these workers, particularly regarding their working conditions, decreased autonomy, compensation schemes or the inherent safety issues and precariousness of gig work [5,15,16,17]. Furthermore, some authors also suggest that gig work is linked with mental health issues [18,19,20,21].

Despite its impact on working conditions in the gig economy, few researchers have looked specifically at algorithmic compensation, which refers to the near-complete automation of the calculation and subsequent granting of monetary rewards for gig work [5,22,23]. Indeed, the literature on this topic rarely distinguishes AM from its compensation component, thus offering little empirical knowledge on the subject [5]. In fact, this distinction is important to properly analyze the effects of algorithmic compensation and gig work. By examining this distinction in this study, we will be able to identify specific impacts, thereby broadening the analytical framework and understanding of gig work.

In particular, AM, including compensation practices, raises issues related to organizational justice, as the automation of managerial decisions to optimize productivity requires a degree of monitoring, control and task division that leaves little room for worker input [24,25,26,27]. For example, in order to assess work performance and calculate pay a number of gig economy online labor platforms (OLP), such as Foodora, Deliveroo and CrowdFlower, collect continuous GPA data, feedback from customers, colleagues and may monitor facial expressions [5,26,28,29]. Given the scope and the impact of these algorithmic decisions on gig workers and their reduced voice and control over these procedures, procedural justice issues seem significant, because employee “voice” shapes procedural justice perceptions. It has been established that this has numerous consequences, notably in respect of occupational health and safety [30,31,32,33]. For example, Magnavita et al. [34] have shown that decreased procedural justice perceptions were related to mental and physical health issues.

Also, algorithmic compensation enables platforms to reward only the “productive” time of task completion which seems to worsen working conditions in gig work, due to the fact that these workers usually perform a number of unpaid related tasks, leading to feelings of stress due to the need to optimize so-called productive moments [24,35,36]. This race for efficiency seems to generate significant stress and work intensification. Indeed, several authors associate gig work and AM to a version of digital Taylorism, with similar practices to those pioneered by the scientific management of the previous century [35,37,38,39]. Despite the proliferation of literature on gig work, few researchers have empirically measured its impact on workers’ health [40,41]. Yet, decades of research on stress have shown across multiple perspectives the detrimental effects of work-related stress and pressure on individuals optimal functioning at work and in general life [42,43].

Furthermore, with the growing number of firms using AM there is a need to understand what may mitigate its detrimental effects on work conditions. Current research indicates that the opacity of operational algorithms, particularly those used for compensation, exacerbates deleterious effects on working conditions [44,45]. Hence, some authors suggest increasing transparency in these algorithmic systems in order to make the mechanisms leading to algorithmic decisions more explainable, thereby enabling workers to have greater voice, more power and, ultimately, more autonomy, thus making working conditions less difficult [5,8,22,24]. However, despite these assumptions, there are few empirical studies that evaluate the extent to which algorithmic transparency mitigates the impacts of AM on work conditions.

Given that the literature on this topic is at an early stage, the aim of this quantitative study is to contribute to a deeper understanding of these novel issues. More precisely, we analyze the influence of algorithmic compensation on gig workers’ perceptions of procedural justice and time-based stress. In addition, we will evaluate the effect of algorithmic transparency as a potential remedy for the impacts associated with algorithmic compensation.

Our paper furthers the understanding in this research area in several ways. First, by examining algorithmic compensation separately from AM, we will attempt to reconcile organizational reality with the literature, while proposing a replicable conceptualization. Also, this study contributes to the emerging thinking on the applicability of perceived organizational justice, particularly its procedural tangent, within the gig economy. Finally, this paper fosters nuance and deepens the knowledge related to the influence of transparency in AM but also to its limitations.

On the practical side, given the explosive growth of the gig economy, this study could contribute to ongoing efforts and discussions to improve working conditions. Our research could detail certain impacts of transparency as well as the influence of algorithmic compensation, potentially helping decision makers, gig workers or platforms examining AM.

This paper is organized as follows. The next section reviews the literature on algorithmic compensation and the other variables of our study, namely procedural justice, time-based stress and algorithmic transparency. The ‘Methodology’ section details our research methods, while the ‘Results’ section displays our findings and states the descriptive statistics in our sample. Then, our ‘Discussion’ section highlights the theoretical and practical contributions of this study, while our final section offers a short conclusion.

## 2. Literature Review

### 2.1. Algorithmic Compensation and Procedural Justice

Organizational justice is defined as the role that fairness plays in employees’ working conditions and perceptions of them [46,47]. Colquitt et al. [48] have shown that in can be modeled in three branches: distributive justice, which focuses on fairness in the allocation of resources; procedural justice, which refers to workers’ perceptions of fairness in the decision-making process leading to resource allocation; and interactional justice, which focuses on the perception of fairness related to individual treatment in work processes [48].

In order to assess procedural justice perceptions, Leventhal’s [49] justice judgment model seems the most generally accepted theoretical conceptualization. In fact, organizational perceptions of procedural justice are based on the degree to which workers consider that organizational procedures meet the six criteria proposed by Leventhal [49], namely, consistency, bias-suppression, accuracy, correctability, representativeness and ethicality. The current literature suggests that algorithmic compensation as perceived by gig workers likely violates several of these rules.

First, OLPs automate performance management by disciplining or rewarding workers, based on consumer ratings [8]. Indeed, gig workers who do not achieve adequate customer satisfaction scores can be penalized, whereas top performer may receive increased rewards [50,51]. However, customers often fail to provide feedback when tasks are completed and gig workers have virtually no opportunity to give their assessment when these automated systems malfunction [51,52]. Therefore, performance management is often evaluated using a curtailed view, as gig workers find themselves without influence on this mechanism. These situations seem to suggest a lack of information accuracy, a criterion in the justice judgment model, which emphasizes the importance of only using complete information in organizational processes. Moreover, several studies report accounts from gig workers highlighting the unfair, subjective and volatile nature of this facet of algorithmic compensation [25,27,36,44]

Second, the weight of customer satisfaction on gig workers’ compensation, which is reflected both in the job evaluation of the service they provided and in tips received, is far from being free of bias. Several authors have raised the sometimes-discriminatory nature of customer evaluations, which are not subject to any verification and thus rely on the everyone’s various biases [51,53,54,55,56]. Furthermore, electronic customer reviews seem prone to be biased by emotional contagion when visible to other customers [57]. This evidence suggests that the weight of customer evaluations in algorithmic compensation is likely to violate the bias-suppression criterion.

Third, this automated client-assessment-dependent compensation is often claimed to be more accurate, as it be less error-prone than traditional appraisal [16,58]. However, when these automated systems malfunction, or when a malicious customer gives a review there is virtually no recourse for gig workers, particularly following a negative evaluation [8,27,59,60]. Many accounts detail that this phenomenon is fraught with consequences, as on the one hand, platforms can unilaterally disable collaboration and thus deprive individuals of their income, and on the other hand, they do not have adequate channels in order to appeal these decisions and restore their employment [27,44,59,60]. These situations seem to run counter to the correctability criterion of Leventhal [49]. In fact, allowing individuals to appeal organizational decisions increases the perceptions of procedural justice.

Fourth, algorithmic compensation mechanisms are subject to unilateral and unannounced changes, as OLPs are private firms with gig workers viewed as contractors, not employees. This situation may lead to significant consequences for these gig workers, who see their wage potential changed from one day to the next, which lead to feelings of apprehension and acts of defiance [27,59,61]. These repeated changes in the retribution mechanisms create unpredictability in algorithmic compensation systems and seem to run counter to the criterion of uniformity and consistency of procedures. Indeed, even if these rules are generally applied uniformly to platform workers, the lack of stability over time can undermine the perceptions of procedural justice.

Fifth, the literature suggests that algorithmic compensation in the gig economy is organized without gig workers’ input, increasing the platforms’ control level and reducing employee “voice” [5,27,52,58,62,63]. Indeed, gig workers have limited means of influencing workflow management, customer reviews or the tipping system. According to Colquitt et al. [64], the criteria of Leventhal [49] and “voice” are the two items most strongly shaping procedural justice perceptions.

By virtue of these arguments, we propose the following hypothesis:

**Hypothesis** **1:** *Algorithmic compensation is negatively related with procedural justice perceptions.*

### 2.2. Algorithmic Compensation and Time-Based Stress

The gig economy offers its workers great flexibility in scheduling, which seems to be the most attractive aspect of gig work, leading to increased job satisfaction [18,65,66,67,68,69]. However, this creates a paradox, as the literature suggests that AM reduces worker’s autonomy due to externally defined goals, rigid time constraints and increased monitoring [5,16,70]. These characteristics of algorithmic compensation appear to accentuate time constrains and intensity workload, which are associated with time-based stress [71,72]. Time-based stress refers to feelings of anxiety related to the increased work demands employees may perceive in having too little time to complete a task [73]. Furthermore, according to several authors, gig work is akin to piece-rate work, an intense form of pay-for-performance [39,58,59,74]. In fact, piece-rate compensation enables OLPs to compensate only “productive” time, which results in gig workers spending long stretches on OLPs unpaid, looking for work, leading to lengthier work hours and the incentive to accept as many gigs as possible, even the disliked or dangerous tasks [24,36].

Also, while meal delivery platforms offer gig workers a flexible schedule, they also constantly measure, guide and limit trips in order to standardize service and may penalize gig workers for not meeting their targets [26,39,65]. Furthermore, these control mechanisms can fail, sending bikers in car-only lanes or missing GPS data preventing workers from receiving their delivery, thereby prolonging the trips and undermining customer satisfaction and productivity goals [26,65,69,75]. While gig workers have limited control vis-à-vis these algorithmic compensation issues, they may still be penalized by them. According to Karasek Jr. [76]’s stress model, this kind of reduced worker autonomy leads to feeling of job stress. Studies have shown that restaurants not having order ready on time, gig workers having their means of transport stolen, customer behaviors and the platform mechanisms themselves can lengthen the time of a task [15,59,69,75,77]. Furthermore, the algorithmically set objectives need to be achieved during bad weather, which increases the risks of accidents [15,77]. Despite these arguments, few researchers seem to have empirically measured algorithmic compensation’s impact on time-based stress and workers’ health [40,41]. Nevertheless, for these reasons, we propose the following hypothesis:

**Hypothesis** **2:** *Algorithmic compensation is positively related to time-based stress.*

### 2.3. The Moderating Role of Algorithmic Transparency

As we noted earlier, the literature regarding transparency in algorithmic compensation appears to be in an early stage and few studies have measured its effects. Nonetheless, the generalized opacity of AM algorithms leads to several issues in the gig economy. Algorithmic transparency refers to the explainability of automated decisions as well as the degree of understanding of individuals towards them [78]. Particularly, according to Malhotra [79], this lack of transparency may exacerbate procedural justice problems in gig work. Indeed, opacity in customer appraisals, tipping mechanisms and changes in algorithmic procedures in ride-sharing platforms could cause AM to negatively impact procedural justice perceptions, thus causing adverse health effects [34,79]. For example, some gig economy firms do not inform gig workers which performance criteria are assessed for increased monetary rewards [40]. This opacity seems to hinder the ability to evaluate the criteria of consistency, bias-suppression, accuracy, correctability and representativeness of Leventhal [49]. Furthermore, the lack of transparency in these automated systems seems to exacerbate the loss of “voice” from algorithmic compensation and increases anxiety related to procedures, because gig workers do not have the tools to understand them and thus cannot offer meaningful input [5,24,27,80,81] Therefore, with greater transparency, gig workers could be informed and able to judge procedures for themselves, seemingly leading to increased procedural justice perceptions.

Also, the opacity of AM systems appears to exacerbate the development of feelings of misunderstanding, uncertainty, frustration, reduced trust and dignity, work overload and stress [5,7,8,52,82,83,84,85]. For example, according to Parent-Rocheleau and Parker [5], a lack of transparency in algorithmic performance management is likely to lead to role ambiguity, as gig workers rarely have explanations about how the algorithms work or how work data are used. A study by Bucher, Schou and Waldkirch [24] points out that gig workers invest considerable time and effort in unpaid tasks, leading to an intensification of workload, as what is measured by the algorithms is unknown, thus the criteria for compensation as well. Furthermore, a lack of algorithmic transparency in automated decision systems prevents its assessment from individuals, who may be inclined to accept every decision, which may lead to work overload and thus feelings of time-based stress [22]. For instance, in ride-sharing platforms, gig workers do not have access to the details of the next ride before accepting it and are subject to time constraints to accept a task, thus preventing a specific evaluation of the following job, they resign themselves to accept every gig offered by these platforms [8,16]. Similarly, algorithmic transparency have been found to foster fairness perceptions and prevent withdrawal intentions among truck drivers [86]. As a result, more transparency in these algorithmic compensation systems could increase decision-making and control for gig workers. Increasing gig workers’ control over their tasks could denote a resource that can alleviate the effects of time-based stress [5,76,87]. Based on the arguments, we propose the following hypothesis:

**Hypothesis** **3:** *Perceived algorithmic transparency will moderate the relationship between algorithmic compensation and procedural justice, such that this relationship will be weaker when perceived transparency is high.*

**Hypothesis** **4:** *Perceived algorithmic transparency will moderate the relationship between algorithmic compensation and time-based stress, such that this relationship will be weaker when perceived transparency is high.*

**Figure 1:**
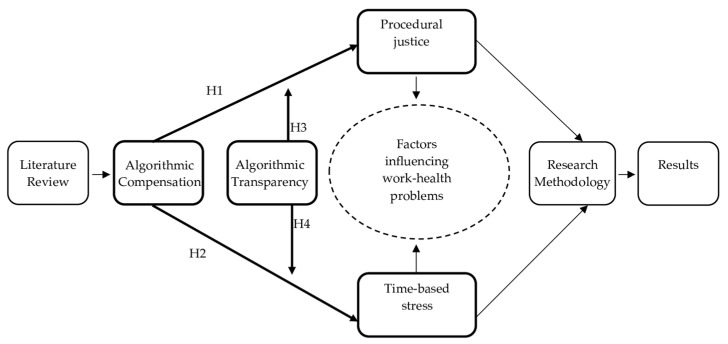
Flowchart of this study showing the process in which algorithmic compensation may influence work-health problems.

In order to illustrate the novelty of our proposed model and emphasize that numerous factors may influence worker health issues, we offer the following flowchart of this study in Figure 1. Furthermore, our hypotheses and variables, concerning the moderating effect of transparency in the relationship between algorithmic compensation and procedural justice are shown in bold.

## 3. Methodology

### 3.1. Participants and Procedure

Our literature review notes a lack of quantitative empirical research on the variables under study and the need to diversify the methodological approaches in this field. With this in mind, we opt for a hypothetical-deductive and quantitative approach in order to test our hypotheses on data collected from gig workers. As part of a larger research project, data collection in our study was conducted using an external research panel. This panel combined social networks, a pre-established bank of respondents and crowd-work platforms, such as Mechanical Turk, in its solicitation for our project. We aimed for a target of 1500 participants.

Participants were paid a fee of USD 5 for participating in the study. A total of 1200 gig workers took part in this study, which led to a final sample of 962, after removing incomplete surveys and participants who failed attention checks [88].

Of the respondents, 59.8% were male, while 38.8% were female, which seems to indicate, similarly with the literature, that there exists a male preponderance in the gig economy [89]. Furthermore, participants were coded in seven age categories, namely under 18 years old (0.1%), between 18 and 24 years old (11.5%), between 25 and 34 years old (36.4%), between 35 and 44 years old (32.2%), between 45 and 54 years old (13.1%), between 55 and 64 years old (5.5%) and between 65 and 74 years old (1.3%). Moreover, 53.6% of respondents worked for crowd-work platforms, like MTurk, whereas 43.2% worked for app-work platforms like Uber. A total of 63.1% of the sample indicated that gig work was not their primary source of income, while this was the case for 36.9% of the respondents. Also, 83.5% of respondents resided in the USA, whereas 13.4% lived in India.

### 3.2. Measures

#### 3.2.1. Perceived Exposure to Algorithmic Compensation

To measure our independent variable, we used the four items of the perceived exposure to algorithmic compensation, as part of the AMQ recently developed and validated by Parent-Rocheleau et al. [90]. Samples items are “A large part of my compensation is determined by an automated system” and “An automated system is responsible for calculating my pay, with no human intervention” [90]. Items were rated using a scale ranging from 1 (strongly disagree) to 7 (strongly agree) [90]. This scale’s Cronbach’s alpha (α) was 0.93.

#### 3.2.2. Procedural Justice

To measure procedural justice perceptions we used the seven indicators (scale by Colquitt [91]. Examples of the items are: “I had influence over the outcomes of those procedures” and “Those [compensation] procedures have been based on accurate information”. Answers were rated using a scale ranging from 1 (strongly disagree) to 7 (strongly agree). This scale’s Cronbach’s alpha (α) was 0.89.

#### 3.2.3. Time-Based Stress

To measure our second dependent variable, we used the four-item scale developed by Kinicki and Vecchio [73]. Examples of the items are: “I have to rush in order to complete my job” and “I am constantly working against the pressure of time”. Many researchers examining time constraints leading to feelings of job stress have used this measurement tool [92,93,94,95]. Moreover, these authors examined this variable across a number of different work environments, suggesting this measurement could also be valid in an atypical workplace as found in the gig economy. Answers were rated using a scale ranging from 1 (strongly disagree) to 7 (strongly agree). This scale’s Cronbach’s alpha (α) was 0.91.

#### 3.2.4. Perceived Algorithmic Transparency

Algorithmic transparency in compensation was measured using four items derived from the scale proposed by Bujold, Parent-Rocheleau and Gaudet [86]. Examples of the items are: “I am aware of how the automated system calculates my remuneration” and “It is easy to predict how much I will receive as a compensation for my work”. Answers were rated using a scale ranging from 1 (strongly disagree) to 7 (strongly agree). This scale’s Cronbach’s alpha (α) was 0.87.

#### 3.2.5. Control Variables

We controlled for four socio-demographic variables (age, gender, platform type, primary source of income) and positive orientation towards technological change, a dimension of positive appraisal toward technological change, as many authors argue these characteristics can influence responses to technology [18,96,97,98,99,100,101].

## 4. Results

### 4.1. Confirmatory Factor Analyses

In order to evaluate the validity of our measurement model, we performed a confirmatory factor analysis (CFA) using AMOS 26.0 software. This consists of estimating the joint effects of our variables allowing to comparison between the selected model and the unobservable models [102,103]. We evaluated the fit of our model using the root mean square error of approximation (RMSEA), the Tucker–Lewis Index (TLI) and the comparative fit index (CFI). For TLI and CFI we regard values over 0.90 as an adequate fit, whereas for RMSEA values under 0.08 would be acceptable and the best cases would be less than 0.05 [103]. Our theoretical four factor model (algorithmic compensation, algorithmic transparency, procedural justice and time-based stress) compared favorably with the other two alternative models tested (algorithmic compensation and transparency paired together, procedural justice and time-based stress) and a one-factor model. Only the four factor model respected the established critical values, namely χ^2^ = 990.5, (219), CFI = 0.93, TLI = 0.91 and RMSEA = 0.07, suggesting a better reliability than our constructs [103,104]. The tree factor model had χ^2^ = 1328.5 (222), CFI = 0.89, TLI = 0.88 and RMSEA = 0.08, and the one factor model resulted in χ^2^ = 3764, (225), CFI = 0.66, TLI = 0.61 and RMSEA = 0.15. In short, the CFA results indicate that our theoretical four-factor model is a statistically preferable choice for the analysis of our constructs.

### 4.2. Descriptive Statistics and Correlations

The descriptive statistics and correlations among the variables of this study can be found in Table 1. We can observe that algorithmic compensation is positively correlated with procedural justice (r = 0.42, *p* < 0.01) and time-based stress (r = 0.29, *p* < 0.01). This result contrasts *a priori* with our first hypothesis, arguing that the relationship with procedural justice is negative. Furthermore, we also performed Harman’s Single Factor Test using SPSS, in order to examine the common variance bias in our research variables, in which our threshold would be a value below 50%. The single factor had a variance = 35.25%, suggesting our instruments should not significantly bias our results [105].

### 4.3. Hypothesis Testing

In order to test our hypotheses, we performed hierarchical regressions on our dependent variables using three separate models. First, we constructed models (1 and 4) with our control variables. Second, we added our independent variables (model 2 and 5). Our last models (3 and 6) consisted of the previous interactions plus our moderator and its interaction term in VI. These results are presented in Table 2.

Hypothesis 1 holds that algorithmic compensation would be negatively related to procedural justice perceptions. Yet, and surprisingly, the results of Model 2 suggest that the relationship between these two variables is positive and significant (β = 0.24, *p* < 0.001). Thus, Hypothesis 1 is rejected.

With respect to our second dependent variable, we proposed in Hypothesis 2 that algorithmic compensation would be positively related to time-based stress. Table 2 shows that this relationship is also positive and significant (β = 0.23, *p* < 0.001). As a result, Hypothesis 2 is supported.

Hypotheses 3 and 4 hold that algorithmic transparency moderates the relationships between the independent variable and the two dependent variables, so that these relationships will be weaker when perceived transparency is high. As can be seen in Table 2, only one of the two interactions tested is significant. Indeed, the moderating effect in the relationship between algorithmic compensation and procedural justice is significant (β = 0.05, *p* < 0.001) and significantly improves the model’s ability to explain the variance in procedural justice (ΔR^2^ = 0.09 ***). In Hypothesis 3, we proposed that increasing algorithmic transparency could mitigate the negative effect of algorithmic compensation on procedural justice perceptions. Instead, it appears to increase the positive relationship between these two variables. Therefore, such a result suggests that our reasoning is not essentially flawed, as the moderating effect appears to explain some of this relationship. Thus, Hypothesis 3 is partially supported.

The interactions of Model 6 in Table 2 predict that time-based stress is not significant nor does it help explain the variance in time stress across individuals. Indeed, it appears that the positive relationship between algorithmic compensation and time-based stress is independent of perceptions of algorithmic transparency, thus Hypothesis 4 is rejected.

**Figure 2:**
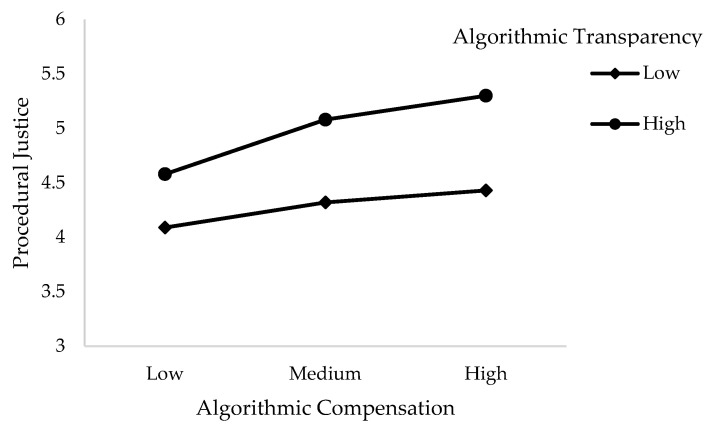
Moderating effect of transparency in the relationship between algorithmic compensation and procedural justice.

Figure 2 consists of simple slopes from our regressions for Hypothesis 3, allowing us to visualize the moderating effects under study. Indeed, even if the coefficient of Hypothesis 3 is low, we can observe that the higher the algorithmic transparency, the more pronounced the relationship between algorithmic compensation and procedural justice.

## 5. Discussion

### 5.1. Theoretical Contributions

This study contributes to the growing literature on the effects of AM, particularly concerning algorithmic compensation. Indeed, few studies have focused on this topic and generally they examine this concept interchangeably with, or as a function of, AM [52,59,80,84]. Indeed, to our knowledge, this study is the first to quantitatively measure the effects algorithmic compensation and its relationship to procedural justice perceptions and time-based stress. Algorithmic transparency has received more attention from researchers, but no study has applied this concept specifically to algorithmic compensation. Indeed, we were able conceptualize it and measure its unexpected impacts, thus opening new avenues, based on more generalizable results.

The results regarding our first hypothesis, which expected a negative relationship between algorithmic compensation and procedural justice, is counterintuitive and thus raise some questions. The positive association between algorithmic compensation and perceived justice of procedures can first be interpreted in the light of specificities of gig work realities. Whereas a vast literature on artificial intelligence (un)fairness highlights the phenomenon of algorithmic aversion and the risks and biases associated with AI tools, another stream of research have started to show that humans may prefer algorithmic decision to human-made decision in certain circumstances and for some types of decisions [106,107,108,109]. We believe that gig workers specifically, because they engage in this work knowing that most of the procedures are technology-mediated and free of human intervention, are likely to assimilate higher level of exposure to algorithmic compensation to greater level of procedural justice. In other words, the justice expectations of gig workers might be higher when everything is automated, whereas human intervention could be perceived as a risk of unfairness.

Relatedly, this adds a question regarding the applicability of the Leventhal [49] criteria, the dominant theoretical framework of procedural justice, to the reality of gig work [110]. Without answering it, highlighting this question sets the table for a potentially important theorical development, knowing that the gig economy’s growth will probably continue in the years to come. Thus, the fact that our quantitative data appear to embolden certain theoretical leads seems to be an interesting contribution to the current literature.

Though, our results are consistent with the hypothesis according to which algorithmic compensation is associated with greater time-based stress for workers. This contributes to the literature on working conditions among OLPs, showing that exposure to algorithmic compensation mode, characterized by piecework pay and algorithmic control over tips and gig allocation is a risk factor for workers. This is because algorithmic compensation forces them to work faster and longer to make a living.

Finally, algorithmic transparency as a field of research is growing rapidly [78,84,111]. On one hand, the results of this study contribute to the advocating for greater explicability in algorithmic decisions by suggesting that transparency fosters greater procedural justice perceptions in algorithmic management. On the other hand, this study also notes that, rather than representing a panacea for all of AM’s ills, transparency fails to mitigate its stressful effect. In other words, algorithmic compensation is stressful, even when the platform provides details and information regarding automated decision-making criteria and processes. First, it could be that some forms of transparency are too cognitively demanding to be helpful to mitigate stress. Second, and worst, it could be that transparency also uncovers information regarding pay procedures that contribute to (rather than reduce) stress. Third, it could also be that or just that it does not change the stressful nature of piecework pay and pay for performance. Thus, our results could benefit future research questions aimed at clarifying the nuanced effect of algorithmic transparency.

### 5.2. Practical Contributions

Our results suggest that transparency has a limited range of applications as a remedy for the consequences of algorithmic compensation. Indeed, our results show that, despite a significant moderating effect in the case of procedural justice perceptions, increasing transparency does not seem to mitigate the time-based stress, due to gig work. Thus, the contribution for actors in these industries is manifold, especially regarding platforms aiming to self-regulate, legislators seeking to build a framework around the use of algorithms in the labor market or platform designs and managers concerned about worker health. Indeed, our results indicate that transparency can be beneficial, but that organizational realities must be considered as a whole in order to maintain a balanced view of the contribution of explicability in an automated system. This result seems to partially nuance the generally accepted view around AM, which offers algorithmic transparency as a solution that could solve most deleterious effect caused by these systems [7,112,113,114]. Thus, to reduce time-based stress, a reevaluation of other aspects of algorithmic compensation, such as pay-for-performance, tip management and the preponderance of customer appraisals, could be needed.

Our study also contributes to the reflections and attempts to regulate gig work. Indeed, our results clearly show that compensation practices are neither necessarily nor fundamentally unfair. Thus, on the one hand, this de-demonization allows us to foresee a great possibility of responsible algorithmic compensation, provided that the practices are supervised. On the other hand, our results also argue for such a framework, given the evidence of the time-based stress experienced by gig workers.

Our contributions also extend beyond the gig economy and are informative for traditional workplaces where AM is implemented. Workers of different sectors with traditional employment arrangement (e.g., transportation, logistics, warehouses, retail, healthcare, factories) are increasingly exposed to AM, including algorithmic compensation practices [22]. Literature reveals similar problems of opacity in algorithmic systems in these settings. Hence, our findings indicate to managers that these practices are seen as stressful and unfair. Albeit incomplete, greater transparency in algorithmic pay determination represent a solution to reestablish the perceived justice surrounding these practices.

### 5.3. Limitations and Future Studies

The first limitation of this study refers to the sampling method, since the participants in the questionnaires could share common traits, thus reducing the generalization of the results. In this case, 83.5% of the respondents were American, and this geographical concentration is a limitation of our thesis, given that the population under study consists mainly of residents of emerging countries [115]. Moreover, these results concerning platform workers seem difficult to generalize to other industries.

Also, a methodological limitation arises from our cross-sectional design, where variables were self-reported and measured in a single questionnaire. This type of design leaves room for common variance bias. Despite our validity analyses and low-to-moderate correlations suggesting that this risk of bias is moderate, studies aiming to replicate our results would greatly benefit from spreading the measures over time and including constructs assessed by third parties. Another methodological limitation of this study is the number of control variables. Given the sample size and the fact that these are new constructs, more control variables, such as trust and job satisfaction, could have better isolated the measured effects.

Moreover, despite interesting results that are at odds with the current literature, several other studies are needed to further investigate this research topic and to ensure the validity of our results. First, it could be rewarding to examine in more detail the effect of transparency as a predictor of procedural justice. Indeed, our results suggest that it acts more as an antecedent than a moderator. Second, it would be interesting to analyze the impacts related to algorithmic compensation and AM of perceived transparency, especially when it reaches high proportions. Indeed, a study on the marginal effect of transparency would allow us to examine certain propositions in the literature, indicating that above a certain level transparency produces unfavorable effects [7,111]. Third, in order to broaden the scope of the research, it would be interesting to conduct a study in relation to the primary source of income and time-based stress. Indeed, our results suggest that this relationship is strong, more so than those found in our hypotheses, and many authors already distinguish these types of gig workers [13,28,100,116]. Thus, future research could investigate the differences between these populations, allowing for an even finer-grained analysis of the effects of algorithmic compensation. Finally, examining other dimensions of organizational justice in relation to algorithmic compensation could be of interest, allowing for a comparison with the surprising results of this research.

## 6. Conclusions

The objective of this study was to analyze the influence of algorithmic compensation on perceptions of procedural justice and time-based stress and to evaluate the moderating effect of transparency in these relationships. The study carried out allowed us to achieve this objective, bringing surprising findings, since our results show that there is a positive relationship between algorithmic compensation and procedural justice and time-based stress. Also, algorithmic transparency mitigates the former relationship, but we did not find an effect on the latter. We believe that this study will deepen the knowledge regarding gig work and new management methods such as algorithmic compensation and management. Thus, our empirical analysis contributes to the evolution of the still early literature on this topic, allowing for the initiation of new avenues for research, and contributes to the development of methods in this industry.

## Figures and Tables

**Table 1:** Descriptive statistics and correlations among research variables.

Variable	M	SD	1	2	3	4	5	6	7	8	9
1. Age	-	-	-								
2. Gender	-	-	0.06	-							
3. Primary source of income	-	-	0.22 **	−0.02	-						
4. Platform type	-	-	−0.11 **	0.00	−0.12 **	-					
5. Positive orientation to technology	5.51	1.13	0.06	−0.14 **	0.04	−0.12 **	-				
6. Algorithmic compensation	4.26	1.82	−0.71 *	−0.06	−0.25 **	0.35 **	0.11 **	-			
7. Algorithmic transparency	5.29	1.38	0.00	−0.03	−0.09 *	−0.31	0.33 **	0.37 **	-		
8. Procedural justice	4.61	1.26	−0.09 **	−0.12 **	−0.16 **	0.14 **	0.39 **	0.42 **	0.47 **	-	
9. Time-based stress	3.71	1.64	−0.18 **	−0.08 *	−0.23 **	0.14 **	−0.80 *	0.29 **	0.07*	0.18 **	-

Notes: N = 962, M = mean; SD = standard deviation; ** = *p* < 0.01; * = *p* < 0.05; age category: 1 = <18 years, 2 = 18 to 24 years, 3 = 25 to 34 years, … 7 = 65 to 74; Sex: 1 = male, 2 = female, 3 = other/neutral; primary source of income: 1 = yes, 2 = no; platform type: 1 = crowd-work, 2 = app-work.

**Table 2:** Results of hierarchical regressions.

Variable	Procedural Justice			Time-Based Stress		
	Model 1	Model 2	Model 3	Model 4	Model 5	Model 6
	β	β	β	β	β	β
Age	−0.08 *	−0.8 *	−0.10 **	−0.19 ***	−0.19 ***	−0.21 **
Gender	−0.18 *	−0.15 *	−0.13	−0.22 *	−0.19	−0.21
Primary source of income	−0.37 ***	−0.19 *	−0.13	−0.62 ***	−0.45 ***	−0.51 ***
Platform type	0.44 ***	0.14	0.21 **	0.33 **	0.04	−0.03
Positive orientation	0.45 ***	0.40 ***	0.32 ***	−0.09 *	−0.15 **	−0.12 *
Algorithmic compensation		0.24 ***	0.16 ***		0.23 ***	0.21 ***
Algorithmic transparency			0.28 ***			0.02
Algorithmic compensation x transparency			0.05 ***			0.00
R^2^	0.22	0.32	0.41	0.09	0.14	0.11
ΔR^2^			0.09 ***			−0.03

Notes: *** = *p* < 0.001; ** = *p* < 0.01; * = *p* < 0.05.

## Data Availability

The data presented in this study are available on request from the corresponding author.
